# Clinical Characteristics of Patients with Dental Malocclusion: An Otolaryngologic Perspective

**DOI:** 10.3390/jcm11216318

**Published:** 2022-10-26

**Authors:** Shin Hyuk Yoo, Ji Hyeok Choi, Ji-Hun Mo

**Affiliations:** Department of Otorhinolaryngology, Dankook University College of Medicine, Cheonan 31116, Korea

**Keywords:** allergic rhinitis, dental malocclusion, orthodontic patients, skin prick test, acoustic rhinometry

## Abstract

**Purpose:** Allergic rhinitis (AR), which is a major cause of upper airway obstruction, may affect the development of the dental malocclusion. This retrospective study was aimed to investigate association between AR and dental malocclusion in otolaryngologic perspectives. **Methods:** Patients (*n* = 217) referred to the otolaryngology department before initiating orthodontic treatment were recruited. The frequency and severity of AR symptoms, sinonasal outcome test (SNOT-22) scores, physical examination findings, acoustic rhinometry results, and treatment modalities were retrospectively assessed. Patients with positive skin prick test findings (SPT) (*n* = 173; orthodontic group) were compared with age- and sex-matched patients being treated for AR (AR group). **Results:** We found that 76.5% of the enrolled patients had subjective nasal symptoms, and 93.1% patients showed abnormal physical examination findings such as inferior turbinate hypertrophy (82.0%), adenotonsillar hypertrophy (31.8%), or deviated nasal septum (7.4%). The 173 (79.7%) patients with positive SPT results exhibited a significantly higher incidence of rhinorrhoea, sneezing, and inferior turbinate hypertrophy compared to those with negative SPT results. The proportion of patients who underwent pharmacological or surgical treatments was significantly higher among patients with nasal obstruction (92.0%) than among patients without nasal obstruction (36.9%). The frequency and mean visual analogue symptom scores for nasal obstruction, rhinorrhoea, and sneezing, as well as all SNOT-22 domain scores, were significantly higher in the AR group than in the orthodontic group. The minimal cross-sectional area measured with acoustic rhinometry showed no significant difference between groups. **Conclusion:** Patients with dental malocclusion had a high SPT (+) rate and a high prevalence of structural abnormalities of the upper airway. The early detection and treatment of subclinical AR, other rhinological problems, and structural abnormalities of the upper airway in patients with malocclusion may help us manage malocclusion from an otolaryngologic perspective.

## 1. Introduction

Craniofacial development is a complex multifactorial phenomenon which can be affected by genetic and environmental factors. As function plays an important role in craniofacial and dental occlusal development [1], previous studies have suggested that prolonged mouth breathing during growth periods of childhood may result in changes in dental and skeletal structures [2,3]. Nasal breathing allows proper growth and development of the craniofacial complex. Mouth breathing is often related to an obstruction in the upper airway, especially in the nasal or pharyngeal regions [4]. Mouth breathing causes postural and structural alterations such as a lower or anterior tongue repositioning of the tongue, open lips, postero-inferior rotation of the mandible, and protrusion of the upper incisors and distal portion of the maxilla [5,6]. Further, changes in the pressure exerted by soft tissues on the teeth and facial bones may also alter these structures. Thus, chronic nasal obstruction is related to craniofacial developmental abnormalities, including temporomandibular joint/dental development disorder, malocclusion, bimaxillary prognathism, and facial asymmetry [7].

The most common cause of mouth breathing is an obstruction of the nasal airway. There are many causes of nasal airway obstruction, including enlarged adenoids and tonsils, deviated nasal septum, sinonasal diseases such as nasal polyps or sinusitis, hypertrophied turbinate, the shape and size of the jaw, and allergic rhinitis (AR).

AR is a major factor affecting the quality of life of children and is a major cause of upper airway obstruction, followed by tonsil and adenoid (T&A) hypertrophy [8,9]. While previous studies have evaluated the effect of AR on the development of dental malocclusion [10,11] and the association between the two diseases from an orthodontic perspective, they have not focused on the otolaryngologic perspective.

## 2. Methods

### 2.1. Patient Enrolment

We retrospectively reviewed the medical records of 217 patients who were referred to the otorhinolaryngology department from the dental hospital before initiating orthodontic treatment between January 2010 and June 2018. From the department of orthodontics, patients requiring treatment for malocclusion at this hospital were referred to the otolaryngology department to rule out any structural abnormalities in the upper airway or AR. Of these patients, 102 patients (47.0%) were male, and 115 patients (53.0%) were female. The age distribution varied from 5 to 29 years (mean age: 13.8 years). According to Angle’s classification of dental malocclusion, 125 patients (57.6%) had type I malocclusion, 74 (34.1%) had type II malocclusion, and 18 (8.3%) had type III malocclusion.

### 2.2. Clinical Course of the Referred Patients

The presence of otolaryngologic symptoms among patients referred from the dental hospital was checked using the Sino-Nasal Outcome Test Score (SNOT-22) questionnaire at the time of the initial visit to the outpatient clinic. Patients were evaluated for abnormalities in the nose and oral cavity through physical and endoscopic examinations by experienced otolaryngologists. The patients also underwent a skin prick test (SPT) and acoustic rhinometry. Patients who complained of otolaryngologic symptoms, as reported in the SNOT-22 questionnaire, were prescribed medications such as antihistamines and decongestants, depending on their symptoms. Patients diagnosed with AR were managed with medications such as antihistamines, intranasal steroid sprays, and leukotriene antagonists in accordance with the Allergic Rhinitis and its Impact on Asthma (ARIA) guidelines [12]. Patients with T&A hypertrophy underwent adenotonsillectomy, and patients with persistent nasal congestion—even those taking appropriate medications—required turbinoplasty. The clinical courses of enrolled patients were investigated through retrospective chart review.

### 2.3. Outcome Measures

#### 2.3.1. Subjective Findings

Subjective parameters including nasal and allergic symptoms such as nasal obstruction, rhinorrhoea, sneezing, and itching were evaluated. Each patient was asked to express their symptoms using a visual analogue scale (VAS) ranging from 0 to 5 (interpretation: 0 = no problem, 1 = very mild problem, 2 = mild or slight problem, 3 = moderate problem, 4 = severe problem, 5 = very severe problem). In addition, all patients scored their symptoms using the SNOT-22 questionnaire at the first outpatient clinic visit. The SNOT-22 scores were analysed using four domains: rhinological (runny nose, need to blow nose, nasal obstruction, thick nasal discharge, loss of smell/taste, postnasal drip, sneezing, and cough), otofacial (ear fullness, ear pain, facial pain/tenderness, and dizziness), quality of life (reduced concentration, difficulty falling asleep, lack of a good night’s sleep, waking up at night, fatigue, reduced productivity, waking up tired), and psychological (frustrated/restless/irritable, embarrassed, sad) [13].

#### 2.3.2. Objective Findings

Physical examination, SPT, and acoustic rhinometry were the objective parameters of assessment. Patients were evaluated for abnormalities in the nose and oral cavity through physical and endoscopic examinations by experienced otolaryngologists. An evaluation of inferior turbinate hypertrophy was based on the degree of obstruction caused by the anterior aspect of the inferior turbinate relative to the total airway space, and was graded as 0–25% (grade I), 26–50% (grade II), 51–75% (grade III), and 76–100% (grade IV) [14]. Grades III and IV were considered to be indicative of inferior turbinate hypertrophy. SPTs were performed with 50 common aeroallergens, including house dust mites, grass, trees, weeds, feathers, cockroaches, cats, dogs, and moulds. All SPTs and their interpretation were performed by experienced personnel. The largest diameter of the wheal and the diameter orthogonal to it were measured for each allergen, and a mean value was calculated. A positive reaction was defined as a mean wheal diameter equal to or greater than that of the positive control (histamine). All the saline controls were negative. Patients were considered allergic if they had at least one positive SPT for any of the allergens tested [15].

The minimal cross-sectional area (MCA) was measured in both pre- and post-decongestant states using acoustic rhinometry (acoustic rhinometer, ECCO vision 3.72, Hood Instruments, Pembroke, MA, USA).

### 2.4. Comparative Analysis with An Age- and Sex-Matched Control Group

The “orthodontic group” included 173 patients (79.7%) with positive SPTs, while a group of 173 age- and sex-matched patients who were being treated for AR in our department were randomly assigned to the “AR” group. The subjective and objective parameters of patients in the two groups were then compared.

### 2.5. Data Analysis, and Statistics

All analyses were performed using the SPSS software (version 22.0; SSPS Inc., IBM Company, Chicago, IL, USA). Pearson’s chi-square test, Fisher’s exact test, and the paired *t*-test were used for statistical analyses. The statistical significance threshold was set at *p* < 0.05.

### 2.6. Ethical Approval and Consent to Participate

This study was approved by the Institutional Review Board of our institution (IRB No. 2021-04-027). Written informed consents were obtained from all participants.

## 3. Results

### 3.1. Demographic and Clinical Characteristics of Patients with Dental Malocclusion

The demographic and clinical characteristics of the enrolled patients are summarised in Table 1. Of the 217 patients, 166 patients (76.5%) had rhinological symptoms: nasal obstruction (*n* = 125, 57.6%), rhinorrhoea (*n* = 104, 47.9%), sneezing (*n* = 82, 37.8%), and itching (*n* = 60, 27.6%). The mean SNOT-22 scores of the patients were 6.8 ± 6.4, 1.8 ± 2.7, 6.5 ± 6.0, and 1.5 ± 2.5 in the rhinological, otofacial, sleep dysfunction, and psychological dysfunction domains, respectively. Physical examination revealed abnormal findings in 202 (93.1%) patients: inferior turbinate hypertrophy (*n* = 178, 82.0%), T&A hypertrophy (*n* = 69, 31.8%), and deviated nasal septum (*n* = 16, 7.4%). A total of 149 (68.7%) patients underwent medical or surgical treatment for rhinological symptoms. While 49 (22.6%) patients received only medical treatment, 100 (46.1%) patients received surgical treatment: adenotonsillectomy (*n* = 21, 9.7%), turbinoplasty (*n* = 30, 13.8%), and adenotonsillectomy with concurrent turbinoplasty (*n* = 49, 22.6%).

**Figure 1:**
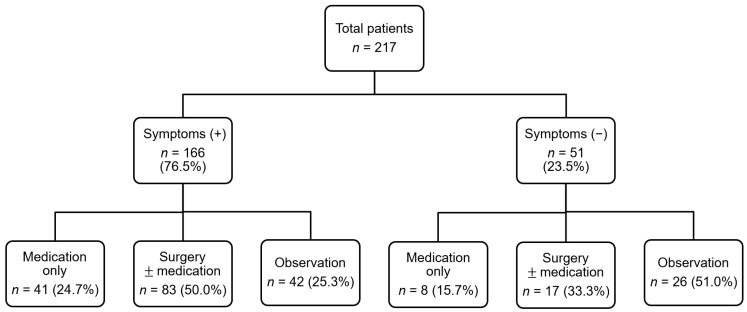
Clinical course of the study participants.

The clinical courses of the study patients are outlined in Figure 1. Of the 166 symptomatic patients, 124 (74.7%) patients underwent medical or surgical treatment, while only 25 (49.0%) patients underwent otolaryngological treatment (Pearson’s chi-square test, *p* < 0.001). However, there were no significant differences in the incidence of positive SPT between symptomatic patients (*n* = 136, 81.9%) and asymptomatic patients (*n* = 37, 72.5%).

As described above, nasal obstruction was the most common symptom of patients with dental malocclusion. Among the 125 patients with nasal obstruction, 115 (92.0%) patients underwent pharmacological or surgical treatment. Of the 92 patients without nasal obstruction, only 34 (36.9%) patients underwent medical or surgical treatment. There was a statistically significant difference in the proportion of patients that received medical or surgical treatment between the two groups (Table 2).

### 3.2. Comparison between Patients with Positive and Negative SPT

Among the 173 (79.7%) SPT (+) patients, 136 (78.6%) patients had rhinological symptoms: nasal airway obstruction (*n* = 101), rhinorrhoea (*n* = 94), sneezing (*n* = 77), and itching (*n* = 52). Of the 44 SPT (−) patients, 30 (68.1%) patients had symptoms of nasal obstruction (*n* = 24), rhinorrhoea (*n* = 10), sneezing (*n* = 5), and itching (*n* = 8).

SPT (+) patients had a significantly higher rate of rhinorrhoea and sneezing than SPT (−) patients (*p* < 0.001). The prevalence of IT hypertrophy was significantly higher in the SPT (+) group than in the SPT (−) group (*p* = 0.007) (Table 3). However, there were no significant differences between the two groups in the prevalence of nasal obstruction, itching, T&A hypertrophy, deviated nasal septum, and medical/surgical treatment rates.

### 3.3. Comparison between Patients in the Orthodontic and AR Groups

**Figure 2:**
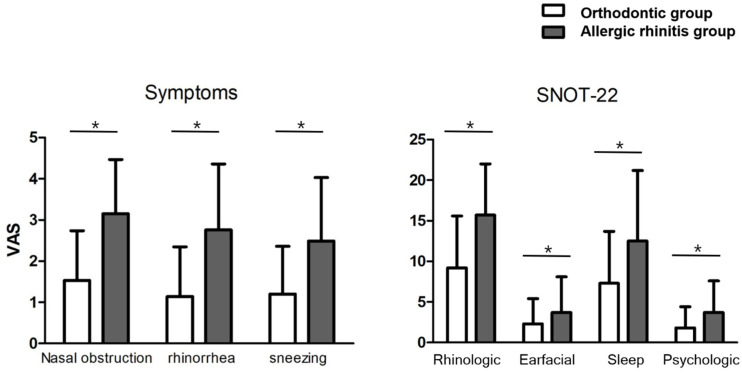
Comparison of visual analogue score (VAS) for symptoms of allergic rhinitis and sino-nasal outcome test score (SNOT-22) between the orthodontic group and the allergic rhinitis group. A paired *t*-test was used to compare the two groups. (* *p* < 0.001).

As described above, 173 patients who were being treated for AR in our department were randomly assigned by age and sex matching and defined as the “AR” group. The subjective and objective assessment parameters of patients from the orthodontic and AR groups were compared (Table 4). The frequency of nasal obstruction (*p* < 0.001), rhinorrhoea (*p* = 0.002), and sneezing (*p* < 0.001) was significantly higher in the AR group than in the orthodontic group. On the other hand, the prevalence of abnormal physical examination findings (T&A hypertrophy, IT hypertrophy, and deviated nasal septum) was not significantly different between the two groups. The mean symptom scores of nasal obstruction, rhinorrhoea, and sneezing were significantly higher in the AR group than in the orthodontic group: 3.0 ± 1.4 vs. 2.0 ± 1.4, 2.5 ± 1.6 vs. 1.5 ± 1.5, and 2.3 ± 1.6 vs. 1.5 ± 1.3, respectively (Figure 2, *p* < 0.001).

The mean SNOT-22 scores of patients in the AR group were significantly higher than those of patients in the orthodontic group in all domains (rhinological: 15.4 ± 6.9 vs. 9.5 ± 6.6, otofacial: 3.4 ± 4.3 vs. 1.9 ± 2.9, sleep: 11.7 ± 8.5 vs. 7.2 ± 6.2, psychological: 3.4 ± 4.0 vs. 1.7 ± 2.7, and total: 39.3 ± 23.6 vs. 23.4 ± 17.0; Figure 2; *p* < 0.001).

**Figure 3:**
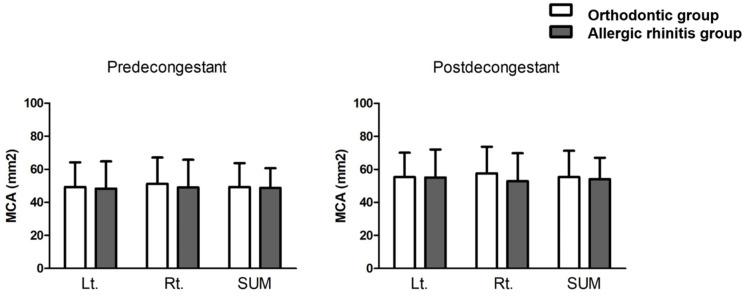
Comparison of minimal cross-sectional areas of nasal cavities measured in pre-decongestant and post-decongestant states between the orthodontic and allergic rhinitis groups. Paired *t*-test was used to compare the two groups. MCA, minimal cross-sectional area; Lt., left; Rt., right; SUM, average value of right and left nasal cavity.

Values related to acoustic rhinometry were also compared between the two groups. The mean MCA values of the right and left nasal cavities in patients from the orthodontic group were 47.9 ± 16.7 mm^2^ and 46.1 ± 16.5 mm^2^, respectively. Following mucosal decongestion, these values changed to 54.0 ± 16.4 mm^2^ and 52.2 ± 16.2 mm^2^, respectively. The mean MCA values of the right and left nasal cavities in patients from the AR group were 45.7 ± 16.3 mm^2^ and 46.1 ± 16.0 mm^2^ in the right and left nasal cavities, respectively. Following mucosal decongestion, these values changed to 51.3 ± 15.8 mm^2^ and 52.4 ± 16.8 mm^2^, respectively. We found no significant difference in the mean MCA values between the two groups, suggesting that patients with dental malocclusion might have structural or constitutional problems of the nasal cavity, although the severity of symptoms was slightly less in the orthodontic group than in the AR group (Figure 3).

## 4. Discussion

In this study, we examined the clinical characteristics of patients with dental malocclusion from an otolaryngologic perspective and evaluated the correlation between AR and dental malocclusion. The incidence of SPT (+) (79.7%), hypertrophied inferior turbinate (82.0%), and T&A hypertrophy (31.8%) in the orthodontic group exceeded the values observed in epidemiological studies in the general population [16,17,18,19]. We found a high prevalence of nasal symptoms and abnormal physical findings even in SPT (−) patients with dental malocclusion. Hypertrophied tonsils, adenoids, and inferior turbinates can cause mouth breathing by obstructing the nasal airway [20,21,22]. These physical findings should be considered as risk factors for dental malocclusion. However, the severity of symptoms was lower among patients in the orthodontic group than those in the AR group. The mean MCA of the nasal cavities measured using acoustic rhinometry was not significantly different between the two groups. These values are similar to those reported in the general population of the same age group in South Korea [23].

Our findings suggest that constitutional or structural problems of upper airway seem to affect the development of dental malocclusions in patients undergoing orthodontic treatment. Upper airway obstruction that induces mouth breathing can lead to the development of malocclusion, by causing an imbalance between the forces from the cheeks and the tongue and an altered position of the mandible [24,25]. Upper airway obstruction and mouth breathing can be induced by many different factors which are responsible for the obstruction of air reflux inside the nasal cavity, deformities of the nasal septum, T&A hypertrophy, nasal polyposis, and AR [26,27].

Asymptomatic AR is believed to lead to persistent inflammation of the nasal mucosa [28]. As this minimal persistent inflammation (MPI) may contribute to co-morbidities of AR such as asthma, it is believed that the treatment strategy may need to include MPI management [29]. For patients with subclinical AR, three major options may be used to treat MPI: antihistamines, anti-leukotrienes, and intranasal corticosteroids [30].

Untreated subclinical AR might contribute to the development of dental malocclusion. Our study suggests that the early detection and treatment of subclinical AR, other rhinological problems, and structural abnormalities of the upper airway in patients with malocclusion may help us manage malocclusion from an otolaryngologic perspective.

Several studies have investigated the association between the type of occlusion and the prevalence of malocclusion in children with nasal obstruction using cephalometric analysis. However, the results of these studies are inconsistent, as the prevalence of malocclusion in children with rhinitis ranges from as high as 78.2% to as low as 3% [31]. Furthermore, few authors have assessed the prevalence of AR and otolaryngologic features in children with dental malocclusion. Luzzi et al. [10] compared 125 patients with malocclusion with 150 healthy patients. They found that children with AR had a three-fold higher risk of developing one or more dento-skeletal alterations. Imbaud et al. [32] evaluated 89 patients who underwent orthodontic treatment and observed a higher prevalence of rhinitis and hypersensitivity to airborne allergens in SPT. However, previous studies, including those described above, did not assess the symptoms or structural abnormalities of AR using quantitative parameters. To our knowledge, this is the first study to compare an orthodontic group with an age- and sex-matched AR group using both subjective and objective quantitative parameters, such as the SNOT-22 questionnaire and acoustic rhinometry. Patients with dental malocclusion in this study had higher prevalences of structural abnormalities on the upper airways and higher rates of SPT (+). Additionally, the results of the acoustic rhinometry showed no differences between the orthodontic group and the AR group. These results imply that structural upper airway abnormalities and subclinical AR may affect dental malocclusion development. Thus, managing dental malocclusions may be assisted by the early detection and treatment of these otolaryngologic problems.

However, the present study has some limitations. Acoustic rhinometry has not yet been accepted as a standard evaluation method because of its unstandardized results [33]. In addition, the results of acoustic rhinometry might be inconsistent due to the nasal cycle, which is a physiological phenomenon that causes a periodic change in nasal airway patency. Thus, future studies must analyse nasal cavity structures using imaging techniques such as computed tomography. In addition, there are various types of malocclusions. Therefore, further research is required to establish the association between AR and the different types of dental malocclusions. Furthermore, a prospective study evaluating the effect of treatment of AR on the development of dental malocclusion is needed to better understand the correlation between AR and dental malocclusion.

## 5. Conclusions

Our study showed higher prevalence of upper airway structural abnormalities and a higher SPT (+) rate among patients with dental malocclusion. The early detection and treatment of subclinical AR, other rhinological problems, and structural abnormalities of the upper airway in patients with malocclusion may help us manage dental malocclusion.

## Figures and Tables

**Table 1:** Demographic and clinical characteristics of patients.

Variable	Total Orthodontic Patients (*n* = 217)
Median age (range), y	13.8 (5–29)
Sex, *n* (%)	
Male	102 (47.0)
Female	115 (53.0)
Symptoms, *n* (%)	166 (76.5)
Nasal obstruction	125 (57.6)
Rhinorrhea	104 (47.9)
Sneezing	82 (37.8)
Itching	60 (27.6)
Sleep disturbance	15 (6.9)
Abnormal PE findings, *n* (%)	202 (93.1)
IT hypertrophy	178 (82.0)
T&A hypertrophy	69 (31.8)
Deviated nasal septum	16 (7.4)
Treatment, *n* (%)	149 (68.7)
Medical treatment	49 (22.6)
Surgical treatment	100 (46.1)
Adenotonsillectomy	21 (9.7)
Turbinoplasty	30 (13.8)
Adenotonsillectomy with turbinoplasty	49 (22.6)

Abbreviations: PE, physical examination; IT, inferior turbinate; T&A, tonsillar and adenoid.

**Table 2:** Treatments performed in patients with and without nasal obstruction.

Variable	Treatment, *n* (%)		No Treatment	*p* ^a^	Odds Ratio
	Medication	Surgery			(95% CI)
Nasal obstruction (+), (*n* = 125)	22 (17.6)	93 (74.4)	10 (8.0)	<0.001	3.7 (2.7–5.1)
Nasal obstruction (–), (*n* = 92)	27 (29.3)	7 (7.6)	58 (63.1)		

^a^ Compared between nasal obstruction (+) and nasal obstruction (−) patients using Pearson’s chi-square test. Abbreviations: CI, confidence interval.

**Table 3:** Comparison of demographic and clinical characteristics between skin prick test (+) and skin prick test (−) patients.

Variable	Total Orthodontic Patients (*n* = 217)		*p* ^a^	Odds Ratio ^a^ (95% CI)
	SPT (+)	SPT (–)		
Total, *n* (%)	173 (79.7)	44 (20.3)		
Rhinologic Sx, *n* (%)	136 (78.6)	30 (68.2)	0.145	1.7 (0.8–3.6)
Nasal obstruction	101 (58.4)	24 (54.5)	0.646	1.2 (0.6–2.3)
Rhinorrhea	94 (54.3)	10 (22.7)	<0.001	4.0 (1.9–8.7)
Sneezing	77 (44.5)	5 (11.4)	<0.001	6.3 (2.4–16.6)
Itching	52 (30.1)	8 (18.2)	0.116	1.9 (0.8–4.4)
Abnormal PE findings, *n* (%)				
IT hypertrophy	148 (85.5)	30 (68.2)	0.007	2.8 (1.3–5.9)
T&A hypertrophy	60 (34.7)	9 (20.5)	0.07	2.1 (0.9–4.6)
Deviated nasal septum	13 (7.5)	3 (6.8)	0.875	1.1 (0.3–4.1)
Treatment, *n* (%)	123 (71.1)	26 (59.1)	0.125	0.6 (0.3–1.2)
Medical treatment	41 (33.7)	8 (18.2)	0.434	1.4 (0.6–3.2)
Surgical treatment	82 (35.8)	18 (40.9)	0.441	1.3 (0.7–2.5)
Adenotonsillectomy	15 (8.7)	6 (13.6)	0.320	0.6 (0.2–1.7)
Turbinoplasty	26 (15.2)	4 (9.1)	0.308	1.8 (0.6–5.4)
Adenotonsillectomy with turbinoplasty	41 (30.4)	8 (18.2)	0.434	1.4 (0.6–3.2)

^a^ Compared between skin prick test (+) orthodontic patients and control group using Pearson’s chi-square test or Fisher’s exact test. Abbreviations: SPT, skin prick test; Sx, symptoms; PE, physical examination; IT, inferior turbinate; T&A, tonsillar and adenoid; CI, confidence interval.

**Table 4:** Comparison of subjective/objective findings between the skin prick test (+) orthodontic group and the allergic rhinitis (control) group.

Variable	Orthodontic Group	Control Group	*p* Value ^a^	Odds Ratio ^a^ (95% CI)
Rhinologic Sx, *n* (%)	136 (78.6)	173 (100)		
Nasal obstruction	101 (58.4)	149 (86.1)	<0.001	4.4 (2.6–7.5)
Rhinorrhea	94 (54.3)	122 (70.5)	0.002	2.0 (1.3–3.1)
Sneezing	77 (44.5)	117 (67.6)	<0.001 ^a^	2.6 (1.7–4.0)
Itching	52 (30.1)	61 (35.3)	0.302	0.8 (0.5–1.2)
Abnormal PE findings, *n* (%)				
IT hypertrophy	148 (85.5)	158 (91.3)	0.093	0.6 (0.3–1.1)
T&A hypertrophy	60 (34.7)	60 (34.7)	1.000	1.0 (0.6–1.6)
Deviated nasal septum	13 (7.5)	22 (12.7)	0.109	0.6 (0.3–1.1)

^a^ Compared between skin prick test (+) orthodontic patients and age- and sex-matched control group using Pearson’s chi-square test. Abbreviations: CI, confidence interval; Sx, symptoms; PE, physical examination; IT, inferior turbinate; T&A, tonsillar and adenoid.

## Data Availability

The data that support the findings of this study are available from the corresponding author, upon reasonable request.

## Abbreviations

AR	allergic rhinitis
ARIA	Allergic Rhinitis and its Impact on Asthma
MPI	minimal persistent inflammation
SNOT-22	sinonasal outcome test-22 questionnaire
SPT	skin prick test
T&A	tonsil and adenoid
VAS	visual analogue scale
